# Supplementary material for: Are carbon emissions trading and green financial instruments synergistic? -Comprehensive quantitative research based on content analysis
Source: PLoS One. 2024 Mar 7;19(3):e0298601. doi: 10.1371/journal.pone.0298601 (PMC10919601; doi:10.1371/journal.pone.0298601)
Supplement: S1 Data — (ZIP) [file pone.0298601.s001.zip › Data/地级市Z_F工作报告.zip!/╕ú╜¿/┴·╤╥╩╨/└┤╫╘╣1⁄2╓┌║┼í╛┬φ┐╦╩2╛▌═°í┐_2018.docx]

各位代表：

　　现在，我代表龙岩市人民政府，向大会报告政府工作，请予审议，并请市政协委员提出意见。

　　一、2017年工作回顾

　　过去一年，在省委、省政府和市委的正确领导下，我市各级政府认真学习贯彻党的十九大精神和习近平新时代中国特色社会主义思想，坚定不移贯彻新发展理念，扎实推进供给侧结构性改革，坚持稳中求进工作总基调，深入打好项目落地、脱贫攻坚、生态环保“三大战役”，着力补齐民生短板，经济社会发展取得新成效。

　　1. 经济发展稳中向好。出台工业21条、服务业20条等产业发展意见及扶持政策，“一企一策”支持龙头企业发展，开展服务业“三比一看”竞赛活动，推动实体经济持续向好。预计全市生产总值2158亿元、增长8%，地方一般公共预算收入138.8亿元、增长5.6%，固定资产投资2519亿元、增长15.1%，规模以上工业增加值635亿元、增长8%。产业结构调整优化，第三产业增加值增长11.4%、对经济增长的贡献率达53%，机械装备、有色金属两大工业主导产业增长20%，高新技术产业增加值增长27%，“八大珍”“八大鲜”、林下经济、花卉苗木、优质果蔬等特色农业加快发展。企业质量效益提升，七成工业企业产值保持两位数增长，企业利润总额增长60%，新增亿元以上企业50家、国家高新技术企业15家，龙工集团成功研发挖掘机高压液压元件，在行业内率先实现国产替代进口，龙工装载机市场占有率稳居行业首位，龙净环保入选工信部制造业单项冠军。新动能不断汇聚，“制造+服务”等新模式进一步推广，龙马环卫获评国家服务型制造示范企业，新培育4家省级服务型制造示范企业。众创、电子商务等新业态加快兴起。军民融合项目新开工16个、新投产9个，国家军民融合新型工业化产业示范基地建设有新进展。成功创建国家应急产业示范基地。

　　2. “三大战役”成效显著。深入开展项目落地攻坚，出台项目工作20条，实行领导挂钩，推进一线考察干部，全力突破征迁难题，“五个一批”项目、重点项目、“重中之重”项目、投资工程包均超额完成年度目标任务，紫金铜业技改扩建、中石油催化裂化剂、龙工“四轮一带”和靖永高速等一批重大项目开工建设或建成投产，杭广高铁双龙段等项目前期取得重大进展。组织开展脱贫攻坚季度攻势行动，突出抓好搬迁扶贫、健康扶贫、金融扶贫等工作，完善精准扶贫信息平台，开展激励性扶贫试点，预计5.7万人如期脱贫。组织实施生态环保“四大提升工程”，全面推行河长制，做好迎接中央环保延伸督察工作，环保突出问题得到有效治理。重点流域1～2类水质比例达36.4%，尤其是中心城区小溪河水质从长期的劣五类提升至三类水标准，登高桥断面部分时段20年来首次达到二类水标准。城市空气质量优良天数比例达99.2%，居全省前列。获评全国森林旅游示范市。龙岩地质公园入选世界地质公园推荐名单。长汀县获评首批国家生态文明建设示范县。

　　3. 城乡建设开创新局面。龙岩市及武平县成功创建全国文明城市。推进创城工作常态长效机制建设，城市管理水平明显提升，城市面貌明显改善，群众共建共享的积极性、主动性大幅提高，市民认同感、获得感明显增强。组织实施中心城区畅通工程，主干道路、拥堵节点改造、停车设施等一批重点城建项目加速推进，全年新开竣工57个项目，新建及改造城市道路17.8公里，新增公共停车位3000多个，新投放一批新能源公共汽车、共享单车，市民出行更加顺畅。制订《龙岩市城市绿化导则》，启动实施中心城市园林绿化提升工程，道路沿线、公园庭院和重要节点绿化改造力度加大，城市绿化美化再上新水平。特色小镇和美丽乡村建设扎实推进，新增1个国家级、2个省级特色小镇和1个田园综合体省级示范点、13个中国传统村落，村镇面貌持续提升。

　　4. 重点改革取得新突破。全国集体林权制度改革现场会在武平县召开，林改“武平经验”进一步推广。漳平市被确定为全国农村集体产权制度改革试点县。顺应“互联网+”发展趋势，建设“e龙岩”网上公共服务平台；开展“减证便民”专项行动，审批服务事项全部进驻网上办事大厅，“一趟不用跑”“最多跑一趟”事项达83.1%，让数据多跑路、百姓少跑腿，群众和企业办事更便捷、更舒心。不动产登记实行“一窗办理、集成服务”，办证速度全省首位、全国领先。全面完成市、县两级政府部门权责清单编制公开工作，基本完成环保机构垂直管理改革，理顺龙岩经济技术开发区、厦龙山海协作经济区和“大古田”管理机制。进一步规范改进市直单位人事编制管理。全面推行预决算公开，加强政府性投资项目概算调整管控。通过政府购买服务方式，发动社会力量参与重点工作。改进中心城区应急性项目建设管理，在“四保”前提下推进城建项目建设提速增效。国有企业改革有序推进，市属国企总资产规模突破1000亿元。创新招商工作机制，坚持领导带队招商，把服务好现有企业作为招商重点，求实求效办好“11·8”大型招商活动，全年新签约项目257个、总投资665亿元。

　　5. 民生保障更加有力。预计城镇居民人均可支配收入32840元、增长8%，农村居民人均可支配收入15655元、增长8.5%；城镇登记失业率2.48%，城镇新增就业1.9万人。全市公共财政用于各类民生支出247.4亿元、增长14%，占地方公共预算支出的82.2%。全面完成23项为民办实事项目，一大批惠民举措落地实施，教育、卫生与健康、养老服务、城乡民生基础设施四大领域补短板工作不断加强。出台加强基础教育工作十条措施，新增中小学学位近1万个、幼儿园学位1.5万个，组建17个教育组团，推行“阳光招生”，推动教育教学质量提升。深化医药卫生体制改革，分级诊疗试点全面推行，妇幼等医疗卫生薄弱领域建设得到加强。提高城乡低保标准，实行城乡居民基本医疗保险市级统筹。基本建成保障性住房1.2万套，新开工建设4080套。成功入选全国居家和社区养老服务改革试点地区。在全国率先通过地方立法保护红色文化遗存，获批设立国家级客家文化（闽西）生态保护实验区。

　　平安县乡村三级联创和“3个10%”做法在全国推广；涉麻制毒、电信诈骗犯罪等专项整治成效显著。安全生产治理能力和监管水平有效提升。食品药品安全放心市建设扎实推进。全民健身和竞技体育全面发展。与此同时，龙岩市及7个县（市、区）被评为全省双拥模范城（县），连续三届“满堂红”，国防动员和后备力量建设成效明显。妇女、儿童、老年人工作继续加强，防灾减灾、对台事务、民族宗教、残疾人、人防、地方志、档案等各项事业取得新进步。

　　6. 政府自身建设不断加强。认真学习贯彻党的十九大精神，深入推进“两学一做”学习教育常态化制度化，在“学懂、弄通、做实”上下功夫，扎实做好政府各项工作。牢固树立“四个意识”，把政治建设摆在首位，坚持在市委统一领导下开展工作。主动接受人大、政协监督和社会监督，办理人大代表建议295件、政协提案645件（次），办复率均为100%，办理质量进一步提升。修订完善《市政府工作规则》，建立市、县两级政府主要领导“三三一”工作机制，制定实施“三大战役”奖励问责办法，增强“马上就办、真抓实干”意识，形成苦干实干狠抓落实的工作氛围。我市机制创新项目在全省绩效管理考评中获第二名。坚持依法行政，政务公开和权力运行网上公开全面推进，“七五”普法深入开展。行政监察、审计监督持续深化，政府系统党风廉政建设和反腐败工作扎实推进。

　　各位代表，过去一年取得的成绩，是坚持以习近平新时代中国特色社会主义思想为指引的结果，是省委、省政府和市委正确领导的结果，是全市人民齐心协力、攻坚克难和各方面大力支持的结果。我代表龙岩市人民政府，向全市人民，向人大代表、政协委员、各民主党派、工商联、各人民团体和各界人士，向中央和省直驻岩机构、驻岩部队和武警官兵，向关心支持龙岩发展的港澳台同胞、海外乡亲和国际友人，表示诚挚感谢！

　　同时，我们更清醒地看到存在的困难和问题，主要是：产业转型提升难度大，经济增长的内生动力不强，发展质量和效益有待提高；优势资源发挥不充分，大项目、好项目缺乏，项目支撑不足；财政增收困难，新的财源点少，地方财力较为薄弱；城乡、区域之间发展差距依然较大，脱贫攻坚仍然艰巨，教育、医疗、养老等民生领域还有许多短板；安全生产、生态环保、社会治安等方面存在不少薄弱环节，基本公共服务和社会保障能力有待进一步提升。政府工作中仍有许多不足，有些政策和改革措施落实不到位，工作机制不完善，一些工作人员的素质、能力和作风不适应新要求，存在担当不够、效率不高、服务不优、工作不实等问题。对此，我们一定直面问题，攻坚克难，做实做好政府工作，推进龙岩加快发展。

　　二、2018年工作安排

　　2018年是贯彻党的十九大精神的开局之年，是改革开放40周年，是决胜全面建成小康社会、实施“十三五”规划承上启下的关键一年。中央、省高度重视闽西老区发展，习近平总书记先后20次到龙岩考察指导工作，先后对长汀水土流失治理、福建集体林权制度改革工作作出重要批示；党的十九大报告特别强调“召开古田全军政治工作会议，恢复和发扬我党我军光荣传统和优良作风，人民军队政治生态得到有效治理”，提出“绿水青山就是金山银山”等新理念。这些为我们加快发展指明了方向，提供了难得机遇，让我们倍受鼓舞、倍感振奋。今后，龙岩红色文化优势和绿色生态优势将更加凸显。

　　我们将在市委统一领导下，全面贯彻党的十九大精神，以习近平新时代中国特色社会主义思想为指导，按照中央“五位一体”总体布局和“四个全面”战略布局，坚持新发展理念，以供给侧结构性改革为主线，坚持稳中求进工作总基调，把实现总量赶超与提高发展质量有机统一起来，统筹推进稳增长、促改革、调结构、惠民生、防风险各项工作，抢抓新机遇，发挥新优势，拓展新资源，深入实施“三大战略”，持续打好“三大战役”，着力产业兴市，着力项目带动，着力城乡协同，着力改善民生，着力改革创新，促进经济社会持续健康发展，推动“再上新台阶、建设新龙岩”迈出新步伐。

　　全年经济社会发展主要预期目标是：全市生产总值增长8.6%；一般公共预算总收入增长3.5%，地方一般公共预算收入增长8.1%；固定资产投资增长15%；外贸出口增速不低于全省平均水平，实际利用外资增长6%；社会消费品零售总额增长12%，居民消费价格总水平涨幅3%左右；城镇登记失业率控制在4.2%以内；城镇居民人均可支配收入增长9.3%，农村居民人均可支配收入增长9.3%；完成节能减排降碳任务。

　　为此，重点做好以下五个方面工作：

　　（一）强化产业兴市，推进发展动力变革

　　落实省“百千万支撑工程”，按照全市“365”产业布局，坚持创新驱动、龙头引领，做强做优做大先进制造业，加快发展现代服务业，壮大实体经济，增强发展内生动力。

　　1. 全力实施“双培育”行动计划。实行“一业一策”“一企一策”，集中优势资源，培育做大支柱产业和龙头企业。培育发展支柱产业。重点抓好有色金属、机械装备、文旅康养三大千亿级产业。围绕打造有色金属之城，推进太阳铜业连铸连轧铜杆、龙德六氟磷酸锂、闪烁及激光晶体等重大项目，延伸拉长金铜循环经济产业链，推动稀土材料开发高附加值产品、拓展应用新领域，力争有色金属产业产值突破700亿元。围绕打造专用车之城，推进新龙马战略重组，加快新能源汽车全产业链发展；抓好环卫保洁、应急电源、排水防涝、冷链物流等专用车项目建设，支持企业从生产领域延伸到工程服务领域，建设省专用车辆产品质量检验中心，做大国家应急产业示范基地。大力推进装载机、挖掘机等整机及配件项目，发展高端工程机械、智能矿山机械等项目，加强大气污染治理设备研制与工程总包，拓展固体废弃物处置、水污染防治等领域产品开发，力争机械装备产业实现产值400亿元以上。围绕打造文旅康养之城、全国性养生养老中心，强化资源整合，引进行业龙头企业，推进体验互动式的业态融合，提升壮大文化旅游产业；发挥独特的红色文化优势，深入挖掘古田会议精神内涵，推进古田红军小镇等项目建设，开发研学旅行精品线路，积极争取国家机关以及央企、部队在岩设立教育培训基地，大力发展红色教育培训产业；深入推进全国居家和社区养老服务改革试点，引进实施一批中高端医养结合项目，打响“清新龙岩·养生福地”品牌，力争文旅康养产业实现产值850亿元以上。同时，继续抓好特色产业发展提升，力争建筑业产值突破900亿元，农产品加工业产值突破400亿元，现代物流业产值突破150亿元，争取成为新的支柱产业。培育壮大龙头企业。完善领导挂钩、部门帮扶、一企一议等机制，在项目推进、用地用林等方面给予倾斜支持，推动紫金矿业、龙烟、龙工、龙净、龙马环卫、金龙稀土、龙洲股份等龙头企业做强做大，推动龙岩高岭土、新罗容和盛等企业尽快上市，加快培育一批产值超10亿、50亿、100亿元企业，打造更多数得上、叫得响的闽西“龙”字头企业军团。支持现有企业技改提升，抓好100个重点技改项目。各县（市、区）和龙岩经济技术开发区均培育1个以上支柱产业和一批龙头企业。

　　2. 积极推动创新融合发展。发挥苏区优势和新古田会议效应，推进军民融合深度发展。持续深化与部队、军工企业的项目、技术、人才、资本对接，加快国家军民融合新型工业化产业示范基地建设，力争全年新开工项目25个以上、新投产项目15个以上、新增25家企业取得参军资质。强化企业主体地位，推进产学研合作，重点推动高端装备制造、新材料、新能源、信息技术、生物医药等领域技术成果引进应用，争取新增国家高新技术企业15家、省级科技小巨人领军企业20家、省级以上研发机构5家，积极创建国家创新型城市。推广服务型制造，支持企业发展主辅分离、个性定制、在线诊断等新模式，新培育一批省级服务型制造示范企业。开展重点行业企业智能制造试点示范，支持企业引进智能制造装备、开发智能终端产品，促进新一代信息技术与制造业加快融合。深化与今日头条、美团点评等互联网企业的对接合作，推动生产、流通、政务、民生等领域大数据普及应用，培育一批创新创业平台，发展新产业、新业态。开展质量提升行动，鼓励企业加强品牌建设，支持重点企业争创制造业“单项冠军”和“专精特新”中小企业。

　　3. 激发各类市场主体活力。大力扶持发展中小微企业。深化成长型中小微工业企业培育计划，继续开展服务业发展竞赛活动，帮助协调资金、技术、用工等问题，支持有市场、有核心技术的企业快速成长，全年新增规模以上工业企业50家、亿元以上工业企业50家、规模以上服务业企业50家。做强做优做大国有企业。深化国资国企改革，加大整合重组，改进经营管理，优化绩效激励，加强风险管控，增强企业投融资能力，加快打造主业突出、规模较大、实力较强的国有企业。鼓励国企开展资本运作，以资源整合、股权合作、资本招商等方式，策划实施优质产业项目，提高国资质量，助推产业转型。激发和保护企业家精神。构建亲清新型政商关系，主动与企业家联系对接，听取企业家建议意见，对突出贡献的企业家进行表彰，形成尊重企业家、支持企业家的良好氛围，鼓励更多社会主体投身创新创业，让市场活力和社会创造力竞相迸发。

　　（二）强化项目带动，推进发展提速提质

　　持续打好项目落地攻坚战，推动“五个一批”项目滚动接续，发挥投资对优化供给结构的重要作用，进一步提高发展的速度和质量。

　　1．全力攻坚重大项目。全年新谋划项目200个、新签约项目200个、新开工项目80个、新投产项目60个、新增资项目50个以上，安排省市重点项目300个，固定资产投资完成2900亿元以上。全力推进60个“重中之重”项目，突出抓好中石油催化裂化剂、龙马环卫装备扩建等30个产业项目，厦蓉高速东联络线、万安溪引水工程等15个基础项目，龙岩大道高架桥等15个城建项目。建成南三龙铁路、厦蓉高速扩容天宝至蛟洋段等项目，力争开工建设杭广高铁双龙段等项目，全力推进龙岩机场、厦渝通道赣龙厦高铁等重大项目前期。

　　2．坚持真招商招真商。主要领导带头招商，实行“管行业必须管招商”，推行全员招商，鼓励以商引商，形成大招商的浓厚氛围。完善招商引资办法，开展常态化、针对性招商，突出主导产业招商，盯紧央企、军工企业，主动对接招商。打好“乡情牌”，实施“龙商回归”工程，吸引龙岩籍企业家返乡投资兴业。既倡导“饿虎扑食”又防止“饥不择食”，坚持优惠与贡献衔接，把好项目准入关，提高招商引资质量。推进龙岩经济技术开发区、厦龙山海协作经济区以及县域特色专业园区建设，完善政策配套和基础设施，做优园区招商平台。主动对接“一带一路”建设，深化龙台产业合作，扩大龙港龙澳合作和国际友城交流，做好新生代侨胞工作，拓宽招商引资渠道。

　　3. 完善项目管理和推进机制。坚持集中精力抓项目，建立完善领导挂钩、定期会商等制度，对“重中之重”项目、“双培育”行动计划项目，成立工作专班，落实“一对一”管理责任，全力攻坚突破。强化项目策划生成，依托支柱产业、龙头企业延伸项目，围绕优势资源开发项目，推动现有企业增资扩产新上项目。强化部门联动，做细做深项目前期，对重特大项目定人、定责、定进度跟踪推进。改革完善项目工程管理制度，推行精细化管理，提高工程效率，确保质量安全，打造精品工程。

　　4．加强要素保障。加大财政资金统筹力度，优化投资计划管理，突出保证重大关键性项目及民生需求迫切项目。用好中央、省支持苏区发展政策，积极向上争取项目资金扶持。完善产融合作平台，促进项目融资精准对接。推进项目测绘、财审、图审等改革，进一步优化审批服务。坚持依法征迁、和谐征迁，集中开展“净地行动”，清理盘活闲置、低效土地，节约集约利用，为新项目提供用地保障。实行差异化扶持政策，集中优势资源推进主导产业、龙头企业项目建设。建立联动执法机制，坚决打击影响施工的各类违法行为，优化项目建设环境。

　　（三）强化城乡协同，推进美丽龙岩建设

　　统筹生产、生活、生态布局，促进城乡基础设施一体化和公共服务均等化，推动人与自然和谐共生，建设宜居宜业的美丽龙岩。

　　1. 推动文明建设常态长效。巩固文明创建成果，着力推进基层行动及群众参与、基础设施提升、政府部门管理、政府服务质量提升、市民素质提高、创城活动运作等常态长效，开展“六项提升行动”，加快形成共建共治共享的城市发展新格局。深化城市执法体制改革，推行“街长”“巷长”负责制，开展无物业小区、农贸市场、夜市、流动摊贩等规范治理，实现城市管理全覆盖。建设新型智慧城市，完善数字城管、智能交通等信息平台，健全巡查报告、受理处置、反馈回访、监督考评等机制，推进城市精细化管理。深化群众性精神文明创建活动，推进诚信建设和志愿服务制度化，进一步增强市民认同感和主人翁意识，让讲文明、守规则、有秩序成为社会风尚。

　　2. 建设宜居宜业城市。认真贯彻“以人为本、尊重自然、传承历史、绿色低碳”的规划理念和“适用、经济、绿色、美观”的建筑方针，突出山水生态元素和历史文脉，推进新一轮城市总体规划修编和“多规合一”成果应用，优化城市设计，彰显特色风貌。在产业协同发展、基础设施一体化建设、公共服务共享等方面加大统筹力度，推动永定区加快融入中心城市。以消除市民出行堵点为目标，继续大力实施畅通工程，加快罗桥节点改造、溪南节点改造、罗龙西路二期、人行过街通道等项目建设，完成“三桥一路”节点改造，火车站北站房综合枢纽建成投入使用，天马路二期、金鸡路全线贯通，全市新增公共停车位2500个以上。推进公交优先发展，更新200辆新能源公交车，新建一批城市公交站场。实施葫芦溪分洪等项目，推进雨污分流和老旧管网改造，进一步提高城市防洪防涝能力。按照“经济、生态、美观”原则，重点抓好龙津湖公园、东山湿地公园建设和龙岩大道一期、大锦山公园等园林绿化提升工程，加快打造景观优美、舒适宜人的城区环境。

　　3. 推进乡村振兴。按照产业兴旺、生态宜居、乡风文明、治理有效、生活富裕的总要求，推进农业农村改革发展。稳定粮食生产，做优七大特色产业，强化农产品质量安全，打响闽西特色农业品牌。培育新型农业经营主体，发展多种形式适度规模经营，提高农业综合效益。大力发展精致农业、休闲农业、农村电商和乡村旅游，构建农村快递物流体系，促进农村一二三产业融合发展，拓宽农民增收渠道。坚持因地制宜、比土不比洋，加快建设2个国家级、5个省级、8个市级特色小镇和永福田园综合体，打造省级美丽乡村120个、美丽乡村景观带3条以上，加强古村落古民居保护。推进健康乡村建设，开展农村人居环境整治行动，抓好农村“厕所革命”和农村危房改造，建立城乡一体化垃圾处理机制，提高农村生活品质。推进“四好农村路”建设，推动农村基础设施提档升级，完成农村路网提级改造450公里、危桥改造60座、农网生命安全防护工程300公里，推行路长制、乡村道路专管员制度，让百姓出行更通畅、更安全。实施一批高效节水灌溉、水库除险加固、农村安全饮水巩固提升等工程，加强农田水利设施建设。抓好农村集体产权制度改革试点，深入推进农村土地“三权”分置和承包经营权确权登记，创新农村“两权”抵押模式，保障农民财产权益，壮大集体经济。做好村级组织换届工作，改革农村“六大员”管理机制，加强农村服务队伍建设，培养造就一支懂农业、爱农村、爱农民的“三农”工作队伍。开展移风易俗行动，提升农民精神风貌，不断提高乡村社会文明程度。

　　4. 加强生态建设。持续打好生态环保攻坚战，守护好、利用好绿水青山这一最宝贵的资源和资产。强化生态环保属地管理、行政执法和企业治污主体责任，推行环境污染责任保险、环保信用评价、信息强制性披露等制度，实行环保督查常态化。推进生态保护红线、永久基本农田、城镇开发边界三条控制线划定工作，做好饮用水源地保护，加强自然保护区、湿地公园建设和管理，加快推进梅花山国家公园前期。打好“清水翻身仗”，总结推广小溪河治理成功经验，深入推进河长制，加大执法问责力度，开展生猪养殖业污染、小流域污染、城市黑臭水体等专项治理，全面消除重点小流域劣五类水质，全市流域水质优良比例达95%以上。打好“青山修复仗”，加大落后产能淘汰力度，加强矿山生态恢复治理，开展国土绿化行动，完成造林绿化11万亩，综合治理水土流失40万亩。打好“蓝天巩固仗”，强化重点工业企业污染治理和臭氧污染防治，确保空气质量保持全省前列。打好“净土保卫仗”，加强铁锰矿、铅锌矿污染整治，推行垃圾废弃物无害化处理，开展化肥农药使用量零增长减量化行动，全面完成土壤污染防治与修复任务。从严治理城市噪音、油污、扬尘等污染，营造良好人居环境。支持企业开展循环经济示范试点，开展创建节约型机关、绿色学校、绿色社区和绿色出行等行动，培育绿色生态文化。

　　（四）强化补齐短板，推进民生事业改善

　　时刻把群众冷暖放在心头，主动回应群众关切，既尽力而为、又量力而行，下大力气解决群众关切的痛点、堵点问题，努力让群众操心事烦心事更少、获得感幸福感更多。

　　1．坚决打赢脱贫攻坚战。精准扶贫是政治责任、是底线任务，我们必须带着情怀和责任抓落实。按照习近平总书记“全面建成小康社会，一个不能少；共同富裕路上，一个不能掉队”的要求，压实责任、精准施策、精细落实，确保在册建档立卡贫困人口全部脱贫、160个贫困村和14个贫困乡镇退出。突出产业帮扶，发挥农业龙头企业带动作用，扶持贫困户发展可直接受益的项目，让贫困户稳定增收。突出金融支持，整合各类扶贫资金，探索建立贫困户资产性收益增收机制。突出健康扶贫，落实好大病医疗补助、医疗叠加保险等政策，加大临时救助、慈善救助等帮扶力度，防止因病致贫返贫。注重扶贫同扶志、扶智相结合，推广激励性扶贫，激发贫困人口内生动力。对无法通过产业扶持和就业帮助脱贫的家庭，实行政策性保障兜底。完善政策引领、投入保障、责任落实、社会参与等脱贫攻坚长效机制，加强脱贫后续保障，提高脱贫质量，确保贫困人口稳定脱贫。用好精准扶贫信息平台，实现全过程指导、推动和监管，以信息化促落实、促公开、促精准，确保脱真贫、真脱贫。严厉查处扶贫领域腐败问题，坚决反对和纠正弄虚作假、做表面文章等侵害群众利益的不正之风。

　　2．促进教育优质均衡发展。坚持把教育事业放在优先位置，努力满足市民对优质教育的新需要。推进城乡义务教育一体化改革发展，努力让每个孩子都能享有公平而有质量的教育。认真落实加强基础教育工作十条措施，全面加强教学质量管理，完善以教绩为导向的职称评聘管、教师教绩考核激励、班主任考评奖励等机制，让一线教师、骨干教师和教学成绩突出的教师“多劳多得、优绩优酬”。加快推进城区基础教育学校扩容，实行代建、施工一体化“交钥匙”工程，全市新改扩建中小学31所、新增学位1万个以上，新办公立幼儿园20所、新增学位7200个以上，扶持发展普惠性民办幼儿园，加快消除“大班额”。深入实施“名校带动工程”，推动基础教育组团发展，扩大优质教育资源覆盖面。深化产教融合、校企合作，推动职业教育转型发展。支持和规范民办教育，办好特殊教育、终身教育，建设学习型社会。

　　3．推进文化繁荣兴盛。坚定文化自信，加快建设文化强市，激发闽西特色文化创新创造活力。培育和践行社会主义核心价值观，传承和弘扬红色文化、客家家风家训，凝聚发展正能量。完善公共文化服务体系，实施文化惠民工程，抓好市博物馆改造提升等项目，多建一批百姓身边的文化设施，丰富群众性文化活动。以国保带省保、县保，加大红色文化遗存保护力度，全面推进国家级客家文化（闽西）生态保护实验区建设。推进海峡两岸交流基地、台湾青年创业基地建设，深化龙台文化交流。规划建设龙岩洞文化创意产业园、特色文化街区，鼓励支持闽西特色文艺创作，大力培育新型文化业态，让文化资源优势转化为产业发展优势，进一步打响闽西文化品牌。

　　4．建设健康龙岩。持续深化医药卫生体制改革，全面推进分级诊疗和医联体实质性运作，加强基层医疗卫生服务体系建设，推动优质医疗资源下沉。加快推进产科、儿科、老年科等薄弱学科建设，新增产科床位100张、儿科床位200张以上。鼓励和支持社会资本办医。精准引进高层次医学人才，定向培养一批本土化医学人才，提高基本医疗服务水平。促进健康与旅游休闲、体育健身、养生养老、中医药等融合发展，实施居民营养计划，研究推广闽西特色药膳养生文化，加快发展健康产业。从严抓好食品药品安全工作，坚决把好百姓饮食用药的每一道关口，创建国家食品安全示范市。继续建设一批市民身边的健身设施，满足市民健身需要。打造“一县一品”全民健身活动品牌，备战第十六届省运会。实施好全面二孩政策，加强与妇幼保健、教育等政策配套衔接。

　　5．健全社会保障体系。就业是最大的民生。实施积极的就业政策，加大对灵活就业、新就业形态的支持，鼓励创业带动就业，抓好高校毕业生、进城务工人员、困难人员等重点群体就业，新增城镇就业1.8万人以上。坚持“房子是用来住的、不是用来炒的”定位，加快建立多主体供应、多渠道保障、租购并举的住房制度。加大棚户区改造力度，基本建成保障性安居工程6000套。完善社会救助体系，继续推进大病统筹保险和医疗救助，继续提高城乡低保标准，扩大低保对象覆盖面，整合资源协力救助在校贫困大学生，实施红土助残工程，使困难群众心里有温暖、生活有奔头。推进社会保险扩面，实施全民参保计划，继续推进机关、事业单位养老保险制度改革。发展社会福利、慈善、老龄事业，关心帮助孤寡老人，支持建设一批居家养老和日间照料中心，加强农村留守儿童关爱保护，切实维护妇女和未成年人合法权益。落实好双拥优抚政策，支持驻岩部队建设，提高国防动员、后备力量和人民防空建设水平。

　　6．建设平安龙岩。平安是发展的前提，是最基本的民生。坚持共建共享，持续深化平安县乡村三级联创活动。推进“互联网+社会治理”，完善立体化、信息化社会治安防控体系，坚决整治群众反映强烈的治安问题。落实综治维稳责任，加大联合惩戒激励力度，提高就地及时解决信访问题能力，筑牢社会稳定的第一道防线。广泛开展普法宣传教育，加强公共法律服务体系建设，积极引导群众养成用法维护权益、解决纷争的习惯。弘扬生命至上、安全第一的思想，认真落实“党政同责、一岗双责、失职追责”，深入开展重点行业、重点领域专项整治，坚决遏制重特大安全事故，提高防灾减灾救灾能力。坚持控新化旧，规范政府融资举债行为，稳妥处置隐性债务。加强金融信用建设，完善不良贷款协调处置机制，依法打击恶意逃废债行为，有效防范涉众型经济风险。依法妥善解决好农民工欠薪问题。创新社区治理模式，推动社会治理重心向基层下移，强化街道、社区治理和服务职能。发挥群团组织在社会共治的积极作用，推进社区、社工和社会组织联动发展。

　　（五）强化改革创新，推进效率变革

　　硬件不足软件补。坚持问题导向、刀刃向内抓改革，做到群众期盼什么、发展需要什么，改革就抓住什么、解决什么，不断增强发展的动力和活力。

　　1．加大“放管服”改革力度。以利企便民促发展为导向，打造行政审批和便民服务升级版，努力让群众、企业办事像“网购”一样方便。完善一口受理、同步审批、限时办结、信息共享等集成审批服务机制，推行“互联网+政务服务”，不断提升“e龙岩”功能。聚焦项目投资、工程建设、社会民生等重点领域，进一步提高审批效率、简化办事程序，推动市级行政权力下放县级管理。加强政府市场监管职能，在更大范围内实行“多证合一”，试行“证照分离”，全面推行网上登记系统，逐步实现电子营业执照跨领域互通互认，提高市场监管和服务水平，以高效便捷的政务服务增进群众认同感、获得感。

　　2. 深化投融资改革。全面实施市场准入负面清单制度，适合市场化运作的建设项目一律向民资开放，鼓励社会资本以特许经营、参股控股等方式，更多投向民生补短板、生态环保等领域。深入推进政银企担合作，推动金融机构支持实体经济发展。大力发展普惠金融，加强对“三农”和小微企业的金融服务。有效运作产业基金，支持企业资本运作，着力解决企业融资难融资贵问题。创新财政支持方式，集中力量扶持重点产业、重点企业发展，加快培育壮大财源。

　　3．创新工作机制。拓展提升水土流失治理“长汀经验”、林改“武平经验”生态品牌，深入开展生态环境损害赔偿制度试点等改革；设立绿色发展基金，探索政府与社会资本合作、第三方治理等模式，鼓励龙净等龙头企业参与水环境治理、固体废物处置，形成更多可推广的龙岩模式，在生态文明试验区建设中走前头。加强对已出台政策措施的跟踪评估，完善抓项目兴产业的常态长效机制。创新引才引智模式，积极引进培育一流人才，推动在岩高校加强应用型办学改革，加快培养一批实用型技能人才。突出在经济建设一线、产业发展中培养和使用人才，充分发挥人才作用。弘扬工匠精神，评选命名一批“龙岩工匠”。完善政府性投资信息系统建设维护机制，推进跨层级、跨地域、跨系统的协同管理和服务。

　　4．严格激励奖惩。落实好“三大战役”奖励问责办法，大力推进一线考察干部，推动正向激励和逆向惩戒常态化。激发县一级作战单元的积极性、创造性，鼓励和支持各级各部门“走出去”跑政策、跑项目、跑资金。完善绩效管理考评办法，全面实行单位内部二级绩效考评，坚持以群众和企业评价结果为依据、以实际效果为导向，倒逼部门提升行政服务效能。坚决整肃懒政怠政行为，确保各项政策和任务不折不扣落到实处。

　　三、进一步加强政府自身建设

　　打铁必须自身硬。我们将深入贯彻习近平新时代中国特色社会主义思想，增强“四个意识”，坚定“四个自信”，坚决维护以习近平同志为核心的党中央权威和集中统一领导，坚持发展第一要务，坚持以人民为中心，增强八种本领，认真履职、积极作为，建设人民满意的服务型政府。

　　1. 建设担当政府。深入开展“不忘初心、牢记使命”主题教育，发扬闽西苏区光荣革命传统和优良作风，拼精神、下狠劲，全力以赴做实做好各项工作。始终以党和人民利益为重，坚决反对当无原则的“老好人”，敢作敢为、担当负责，只要是有利于闽西长远发展的事、有利于群众利益的事，都要紧盯不放、坚持不懈往前做。坚持严管与厚爱结合、激励与约束并重，旗帜鲜明为干事者鼓劲、为担当者撑腰，让广大干部想干事、敢干事、干成事。

　　2．建设法治政府。坚持在党的领导下、在法治轨道上开展工作，推动形成办事依法、遇事找法、解决问题用法、化解矛盾靠法的良好法治环境。自觉接受人大法律监督和工作监督、政协民主监督、监察机关监督，主动接受社会监督和舆论监督。构建依法行政制度体系，完善权力、责任、监管三张清单，持续深化政务公开，严格规范公正文明执法。加强行政执法与刑事司法相衔接。完善风险评估、专家论证、集体研究、公众参与的重大决策机制，广泛听取各方面意见，提高行政决策科学化、民主化水平。

　　3. 建设实干政府。不驰于空想，不骛于虚声。坚持“实”字为本，说实话、谋实事、出实招、求实效，把雷厉风行和久久为功有机结合起来，多干少说、力戒空谈、反对“作秀表演”，以求实的思维多谋打基础、利长远之策，以务实的行动多做破难题、解民忧之事。认真办好市委、市政府28件为民办实事项目，着力解决群众需求最迫切的问题。坚持“干”字当头，埋头苦干、攻坚克难，当好“施工队长”，一件接着一件干，以钉钉子精神抓好工作落实，把好思路定成好机制、好方案做成好项目，以好项目推动高质量发展。坚持“新”字为要，创新机制方法，善于运用互联网技术和信息化手段开展工作，推动社会治理精准化、公共服务高效化。纠正文山会海，改进检查评比方式，提高督查督办质量，减轻基层负担。

　　4. 建设廉洁政府。认真落实新时代党的建设总要求，深入推进党风廉政建设和反腐败斗争。巩固拓展落实中央八项规定精神成果和市委《关于在全市党员干部中纠正不良习气树立清风正气的意见》，锲而不舍改进作风，坚决反对和纠正形式主义、官僚主义。紧紧扎牢制度的笼子，依法依规加强对公共资金、公共资源、人事编制、国有资产等监管。推进审计管理体制改革，更好发挥审计监督作用。推行政府购买服务，把第三方可提供的事务性管理服务交给社会去办。坚持过紧日子思想，把每一笔钱花在明处、用在实处，进一步压缩一般性支出，集中更多财力用于发展产业、改善民生。

　　各位代表，幸福都是奋斗出来的。新时代、新使命，新征程、新作为。让我们在习近平新时代中国特色社会主义思想指引下，更加紧密团结在以习近平同志为核心的党中央周围，在中共龙岩市委的坚强领导下，不忘初心、牢记使命，扑下身子抓落实、心无旁骛促发展，为决胜全面建成小康社会，加快建设机制活、产业优、百姓富、生态美的新龙岩而不懈奋斗！
